# The Effects of Implementing a Mobile Health–Technology Supported Pathway on Atrial Fibrillation–Related Adverse Events Among Patients With Multimorbidity: The mAFA-II Randomized Clinical Trial

**DOI:** 10.1001/jamanetworkopen.2021.40071

**Published:** 2021-12-21

**Authors:** Yuan Yao, Yutao Guo, Gregory Y. H. Lip

**Affiliations:** 1 Institute for Hospital Management Research, Chinese PLA General Hospital, Beijing, China; 2 Department of Pulmonary Vessel and Thrombotic Disease, Medical School of Chinese PLA, Chinese PLA General Hospital, Beijing, China; 3 Liverpool Centre for Cardiovascular Sciences, University of Liverpool, Liverpool Heart & Chest Hospital, Liverpool, United Kingdom; 4 Aalborg Thrombosis Research Unit, Department of Clinical Medicine, Aalborg University, Aalborg, Denmark

## Abstract

**Importance:**

The Mobile Health Technology for Improved Screening and Optimized Integrated Care in Atrial Fibrillation (mAFA-II) trial is a prospective cluster randomized trial that found a significant reduction in the composite clinical outcome of stroke or thromboembolism, all-cause death, and rehospitalization among patients with atrial fibrillation (AF) who used a mobile health (mHealth) technology that implemented the Atrial Fibrillation Better Care (ABC) pathway (ie, A, anticoagulation/avoid stroke; B, better symptom control; and C, cardiovascular disease and comorbidity management) compared with those receiving usual care. Multimorbidity (defined as ≥2 chronic long-term conditions) is common in older patients with AF, but the impact of integrated or holistic care (based on the ABC pathway) on clinical outcomes in this population is uncertain.

**Objective:**

To evaluate whether implementation of the integrated ABC pathway, supported by mHealth technology, would reduce AF-related adverse events in patients with multimorbidity.

**Design, Setting, and Participants:**

This prespecified ancillary analysis of data from the extended follow-up of the mAFA II trial was conducted between June 2018 and April 2021. Adult patients with AF were included in the analysis if they had at least 2 comorbidities. Participants were enrolled across 40 centers in China.

**Intervention:**

Integrated care supported by mHealth technology (mAFA intervention) vs usual care.

**Main Outcomes and Measures:**

The main outcome was the composite outcome of stroke or thromboembolism, all-cause death, and rehospitalization. Cox proportional hazard modeling was performed for adverse outcomes after adjusting for cluster effect and baseline risk factors.

**Results:**

Of 1890 patients, 833 (mean [SD] age, 72.0 [12.0] years; 278 [33.4%] women) with multimorbidity were allocated to the intervention group (ABC pathway), with a mean (SD) follow-up of 419 (257) days, and 1057 patients (mean [SD] age, 72.8 [13.0] years; 443 [41.9%] women) with multimorbidity were allocated to usual care, with a mean (SD) follow-up of 457 (154) days. Compared with usual care, the composite outcome of stroke or thromboembolism, all-cause death, and rehospitalization was significantly reduced in the intervention group (hazard ratio [HR], 0.37; 95% CI, 0.26-0.53; *P* < .001), as were rehospitalizations alone (HR, 0.42; 95% CI, 0.27-0.64; *P* < .001). For the C criterion of the ABC pathway, rates of acute coronary syndrome, heart failure, and uncontrolled blood pressure during follow-up were lower in the intervention group than the usual care group (27 patients [3.2%] vs 145 patients [13.7%]; HR, 0.29; 95% CI, 0.19-0.45; *P* < .001). Subgroup analyses by age, prior stroke, and sex demonstrated consistently lower HRs for the primary composite outcome and rehospitalization for patients with AF allocated to the intervention group compared with patients receiving usual care.

**Conclusions and Relevance:**

In this study, mHealth technology–based integrated care that facilitated the implementation of the ABC pathway reduced meaningful clinical adverse events in older patients with AF and multimorbidity vs usual care.

**Trial Registration:**

WHO International Clinical Trials Registry Platform (ICTRP) Registration number: ChiCTR-OOC-17014138

## Key Points

**Question:**

Does implementing mobile health technology–supported integrated care reduce atrial fibrillation (AF)–related adverse events in patients with multimorbidity?

**Findings:**

In this prespecified ancillary analysis of data that included 1890 adults with AF and multimorbidity, mobile health–supported integrated care reduced the composite outcome of stroke or thromboembolism, all-cause death, and rehospitalization, irrespective of age, sex, and prior stroke.

**Meaning:**

In this study, an integrated care approach supported by mobile health technology reduced cardiovascular adverse events for older patients with AF and multimorbidity.

## Teaser

This randomized clinical trial evaluates whether implementation of the integrated Atrial Fibrillation Better Care (ABC) pathway, supported by mobile health technology, reduces atrial fibrillation–related adverse events in patients with multimorbidity.

## Introduction

Atrial fibrillation (AF) is the most common type of cardiac arrhythmia worldwide, and it is associated with many other cardiac and noncardiac chronic conditions. For example, one-third of patients with AF have at least 3 associated chronic diseases, including cancer, hematological disorders, and immunological disorders.^1^ With AF, we are also dealing with a common chronic condition in which associated comorbidities lead to higher risks of hospitalization, impaired quality of life, the development of heart failure, and dementia. In a retrospective analysis of the AF Follow-up Investigation of Rhythm Management (AFFIRM) trial, multimorbidity (AF plus ≥2 chronic long-term conditions) was present in 54.4% of patients.^2^ Appropriate AF care requires attention to comorbidities associated with AF, which often occur in clusters rather than as isolated risk factors.

The various AF-related clinical sequelae, symptoms, and complications have a major impact on health care resources.^3^ A considerable part of the health care budget is used by noncardiovascular hospitalizations, which have remained almost unchanged over the years and are likely to increase substantially over the next decades.^4^ The high multimorbidity associated with AF also has implications for uptake of evidence-based therapies, eg, stroke prevention, despite their higher inherent cardiovascular and stroke risks.^5^ Unsurprisingly, the number and types of multimorbidity in patients with AF has been linked to worse outcomes, including higher incidence of stroke, death, and major bleeding.^5,6^

The Mobile Health Technology for Improved Screening and Optimized Integrated Care in AF (mAFA-II) trial was a prospective cluster randomized trial that investigated the implementation of holistic or integrated care in patients with AF using mobile health (mHealth) technology implementing the AF Better Care (ABC) pathway,^7^ in which A indicates anticoagulation/avoid stroke; B, better symptom control; and C, cardiovascular disease and comorbidity management. In the primary analysis of the mAFA-II trial, the rates of the composite outcome of ischemic stroke or systemic thromboembolism, death, and rehospitalization were lower in the mAFA intervention group compared with the usual care group.^8^ The mAFA program also included awareness, screening, and detection of AF (the Huawei Heart Study),^9,10^ followed by a structured management program. This program required used of a mobile application installed on a smartphone and interactions with the mAFA network of researchers to implement the ABC pathway.

Given that multimorbidity (defined as ≥2 chronic long-term conditions) is common in older patients with AF, the impact of mHealth-facilitated holistic or integrated care on clinical outcomes is uncertain. The objective of the present ancillary analysis from continued follow-up of the mAFA-II trial was to evaluate whether implementation of an mHealth technology–supported ABC Pathway would reduce AF-related adverse events in patients with multimorbidity.

## Methods

The design and results of the mAFA-II trial have been previously published.^8,10^ The trial protocol appears in Supplement 1. The study was approved by the Central Medical Ethic Committee of Chinese PLA General Hospital and by local institutional review boards. The study followed Consolidated Standards of Reporting Trials (CONSORT) reporting guideline and complied with the Declaration of Helsinki,^11^ and all patients gave written informed consent. In brief, 1646 patients with AF were allocated to the mAFA intervention (mean age, 67.0 years; 38.0% women) and compared with 1678 patients allocated to usual care. Rates of the composite outcome of ischemic stroke or systemic thromboembolism, death, and rehospitalization were significantly lower with the mAFA intervention compared with usual care (hazard ratio [HR], 0.39; 95% CI, 0.22-0.67; *P* < .001), largely because of lower hospitalization rates.^8^ Patients aged 18 years or older, diagnosed with new-onset, paroxysmal, persistent, or permanent AF confirmed with electrocardiogram or 24-hour Holter monitors were included, while patients younger than 18 years of age, those with mechanical prosthetic valve or moderate or severe mitral stenosis, those unable to provide informed consent, or those unable to be followed up for 1 year for any reason were excluded.

**Figure 1.**
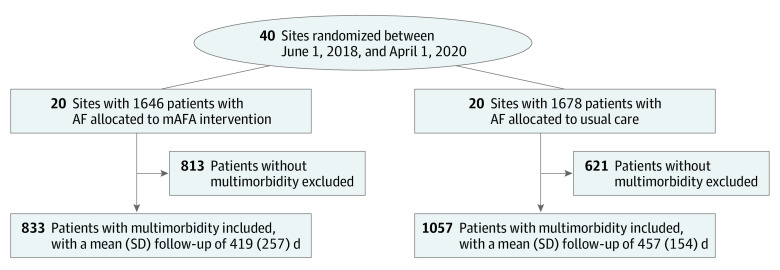
Flowchart of Patients Included in the Mobile Health Technology for Improved Screening and Optimized Integrated Care in Atrial Fibrillation Trial Included participants were patients with atrial fibrillation (AF) and multimorbidity, ie, at least 2 comorbidities, including hypertension, coronary artery disease, heart failure, cardiomyopathy, peripheral arterial disease, diabetes, liver or kidney dysfunction, pulmonary disease, and prior stroke. mAFA indicates mobile atrial fibrillation application.

To investigate the value of using the ABC pathway mAFA intervention in AF patients with multimorbidity (≥2 comorbidities, including hypertension, coronary artery disease, heart failure [HF], cardiomyopathy, peripheral arterial disease, diabetes, liver or kidney dysfunction, pulmonary disease, prior stroke), we studied those participating in the mAFA-II trial between June 1, 2018, and April 1, 2020, across 40 centers in China (Figure 1). For the mHealth-supported ABC pathway, we defined the components as follows: the A-criterion intervention was performed through regular stroke and bleeding risk assessment and reassessment, dose adjustments based on dynamic evaluation of kidney and liver function, and changes in therapeutic range if appropriate. The B-criterion intervention was carried out with patient-centered symptom-directed management, and AF symptoms were evaluated using the European Heart Rhythm Association classification and assessment.^12^ Antiarrhythmic drugs or rate control therapies based on guidelines on AF management were proposed based on patient’s reported AF symptoms. Third, the C-criterion intervention was achieved through proactive management of comorbidities, eg, optimized treatment on monitoring blood pressure. Patients in the usual care clusters received management based on local clinical practice.

### Statistical Analysis

Baseline characteristics for continuous variables are summarized as means and SDs. Frequencies and percentages per group as well as HRs with 95% CIs are reported for binary outcomes. We used Cox proportional hazard modeling after adjusting for cluster effect and baseline risk factors to analyze the primary composite outcome of stroke or thromboembolism, all-cause death, and rehospitalization.

Outcomes in relation to components of the ABC Pathway were assessed as follows. For the A criterion, thromboembolism (ischemic stroke and other systemic thromboembolism) and bleeding events (intracranial bleeding and extracranial bleeding) were analyzed, after adjustment of baseline risk factors. Extracranial bleeding events included gastrointestinal, urogenital, skin, eye bleeding, and other nonmajor bleeding. For the B criterion, recurrent AF and its related symptoms (eg, palpitation) were assessed after adjustment of baseline risk factors. For the C criterion, the occurrence of acute coronary syndromes, HF, and uncontrolled blood pressure during the follow-up period was assessed, after adjusting confounders. Subgroup analyses for the primary composite outcome and secondary outcomes (thromboembolism) were conducted by age, sex, and prior stroke (as secondary stroke prevention) after adjusting for baseline risk factors.

All statistical tests were done using the nominal *P* < .05 significance level, and tests were 2-tailed. All statistical analyses were conducted using SPSS statistical software version 22.0 (IBM Corp) and MedCalc version 19.0.4 (MedCalc Software).

## Results

Of 1890 patients, 833 patients (mean [SD] age, 72.0 [12.0] years; 278 [33.4%] women) with multimorbidity were allocated to the intervention group (ABC pathway), with a mean (SD) follow-up of 419 (257) days (median [IQR], 309 [203-763] days), and 1057 patients (mean [SD] age 72.8 [13.0] years; 443 [41.9%] women) with multimorbidity were allocated to usual care, with a mean (SD) follow-up of 457 (154) days (median [IQR], 478 [355-561] days). Baseline characteristics are shown in Table 1, and treatments by group appear in the eTable in Supplement 2.

**Table 1.** Baseline Characteristics of Patients With AF and at Least 2 Comorbidities Included in the mAFA Intervention and Usual Care Groups

Characteristic	Patients, No. (%)	
	mAFA intervention (n = 833)	Usual care (n = 1057)
Age, mean (SD), y	72.0 (12.0)	72.8 (13.0)
Women	278 (33.4)	443 (41.9)
Men	555 (67.6)	667 (58.1)
Current smoking	102 (12.2)	139 (13.2)
Medical history		
Hypertension	676 (81.1)	797 (75.4)
Systolic blood pressure >160 mm Hg	95 (11.4)	91 (8.6)
CAD	523 (62.8)	763 (72.2)
Diabetes	331 (39.7)	363 (34.3)
Pulmonary disease^a^	266 (31.9)	274 (25.9)
PAD	238 (28.6)	200 (18.9)
Prior ischemic stroke or other TE	229 (27.5)	338 (32.0)
Heart failure	161 (19.3)	215 (20.3)
Prior bleeding	80 (9.6)	86 (8.1)
Dilated or hypertrophic cardiomyopathy	45 (5.4)	67 (6.3)
AF type		
New onset	85 (10.2)	71 (6.7)
Paroxysmal	394 (47.3)	351 (33.2)
Persistent	210 (25.2)	348 (32.9)
Long-standing	24 (2.9)	134 (12.7)
Permanent	27 (3.2)	112 (10.6)
Unknown	93 (11.2)	41 (3.9)
Prior AF treatment		
Pharmacy cardioversion	204 (24.5)	123 (11.6)
Electrical cardioversion	15 (1.8)	11 (1.0)
AF ablation	110 (13.2)	81 (7.7)
CHA_2_DS_2_-VASc score, mean (SD)	3.9 (1.7)	4.0 (1.6)
HAS-BLED score, mean (SD)	1.6 (1.0)	1.7 (1.0)

Abbreviations: AF, atrial fibrillation; CAD, coronary artery disease; CHA_2_DS_2_-VASc, chronic heart failure, hypertension, older than 75 years, diabetes, stroke, vascular disease, aged 65 to 74 years, sex; HAS-BLED, hypertension, abnormal kidney/liver function, stroke, bleeding history or predisposition, labile international normalized ratio, elderly, drugs/alcohol concomitantly; mAFA, mobile atrial fibrillation application; PAD, peripheral arterial disease; TE, thromboembolism.

^a^ Pulmonary diseases include obstructive sleep apnea hypopnea syndrome, chronic obstructive pulmonary disease, and pulmonary hypertension.

**Figure 2.**
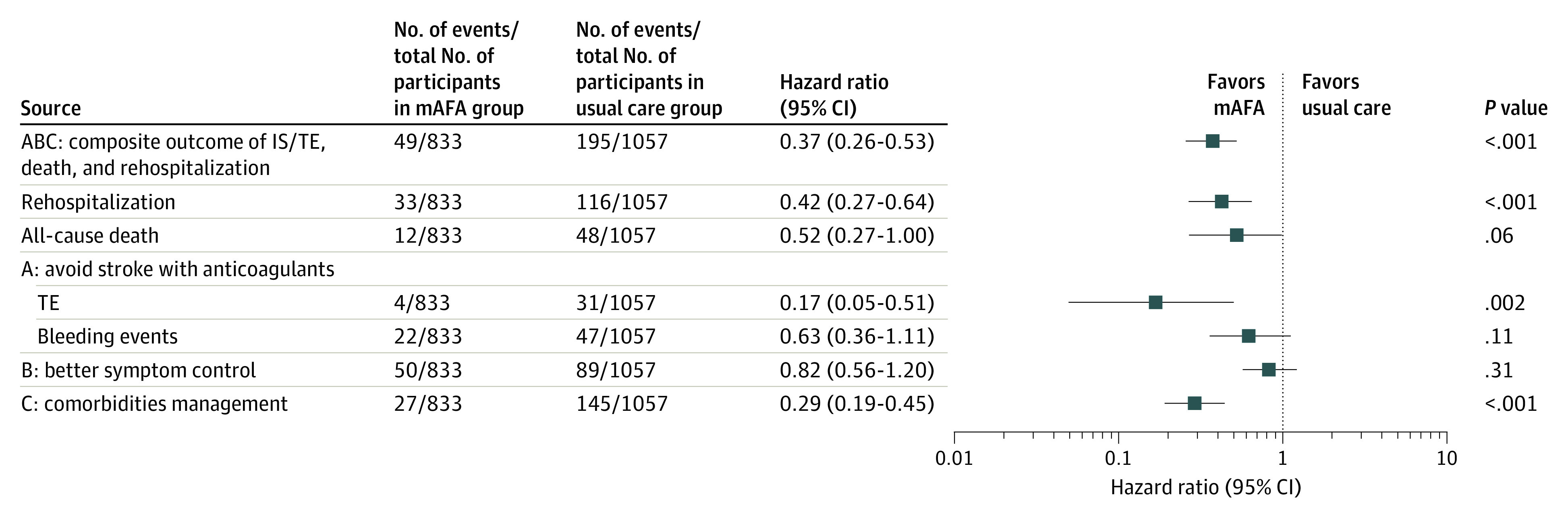
Hazard Ratios of Clinical Events, Adjusted for Baseline Risk Factors B criterion was assessed by reported recurrent atrial fibrillation symptoms, eg, palpitations. C criterion was assessed by the occurrence of acute coronary syndrome, heart failure, or uncontrolled blood pressure during the follow-up period. IS indicates ischemic stroke; mAFA, mobile atrial fibrillation application; and TE, thromboembolism.

Compared with usual care, the composite outcome of stroke or thromboembolism, all-cause death, and rehospitalization was significantly reduced in the intervention group compared with the usual care group (HR, 0.37; 95% CI, 0.26-0.53; *P* < .001), as were rehospitalizations alone (HR, 0.42; 95% CI, 0.27-0.64; *P* < .001). The difference between groups for all-cause death was not statistically significant (HR, 0.52; 95% CI, 0.27-1.00; *P* = .06) (Table 2 and Figure 2).

**Table 2.** Clinical Outcomes in the mAFA and Usual Care Groups

Outcome	Patients, No. (%)		mAFA vs usual care, HR (95% CI)^a^	*P* value
	mAFA (n = 833)	Usual care (n = 1057)		
A criterion: anticoagulation/avoid stroke				
Thromboembolism				
Any	4 (0.5)	31 (2.9)	0.17 (0.05-0.51)	.002
Ischemic stroke	4 (0.5)	11 (1.0)	0.54 (0.16-1.85)	.33
Other systemic thromboembolism	0	20 (1.9)	NA	NA
Bleeding events				
Any	22 (2.6)	47 (4.4)	0.63 (0.36-1.11)	.11
Intracranial	0	9 (0.8)	NA	NA
Extracranial^b^	22 (2.6)	38 (3.6)	0.78 (0.44-1.40)	.41
B criterion: better symptom control				
Reported recurrent AF symptoms, eg, palpitations	50 (6.0)	89 (8.4)	0.82 (0.56-1.20)	.31
C criterion: cardiovascular risk factor and comorbidity management				
Composite of acute coronary syndrome, HF, and uncontrolled blood pressure	27 (3.2)	145 (13.7)	0.29 (0.19-0.45)	<.001
All-cause death	12 (1.4)	48 (4.5)	0.52 (0.27-1.00)	.06
Rehospitalization^c^	33 (4.0)	116 (11.0)	0.42 (0.27-0.64)	<.001
Composite outcome of ischemic stroke or thromboembolism, death, and rehospitalization	49 (5.9)	195 (18.4)	0.37 (0.26-0.53)	<.001

Abbreviations: AF, atrial fibrillation; HF, heart failure; HR, hazard ratio; mAFA, mobile atrial fibrillation application; NA, not applicable.

^a^ After adjustment of cluster effect, age, comorbidities, AF type, and prior AF treatment, the effect of mAFA intervention on the clinical events was assessed.

^b^ Extracranial bleeding included gastrointestinal, urogenital, skin, eye bleeding, and other nonmajor bleeding.

^c^ Reasons for rehospitalization included any cause for AF, HF, thromboembolism, major bleeding, artery coronary disease, and other cardiovascular disease.

Rates of stroke or thromboembolism were lower with the mAFA intervention vs usual care (4 [0.5%] vs 31 [2.9%]; HR, 0.17; 95% CI, 0.05-0.51; *P* = .002), with no significant difference in bleeding (HR, 0.63; 95% CI, 0.36-1.11; *P* = .11) (Table 2). The mAFA intervention led to less frequent occurrence of acute coronary syndrome, HF, and uncontrolled blood pressure during the follow-up period (27 [3.2%] vs 145 [13.7%], HR, 0.29; 95% CI, 0.19-0.45; *P* < .001) (Table 2). There were no significant differences in AF symptoms between mAFA and usual care in this group with multimorbidity (Table 2).

### Subgroup Analyses

**Figure 3.**
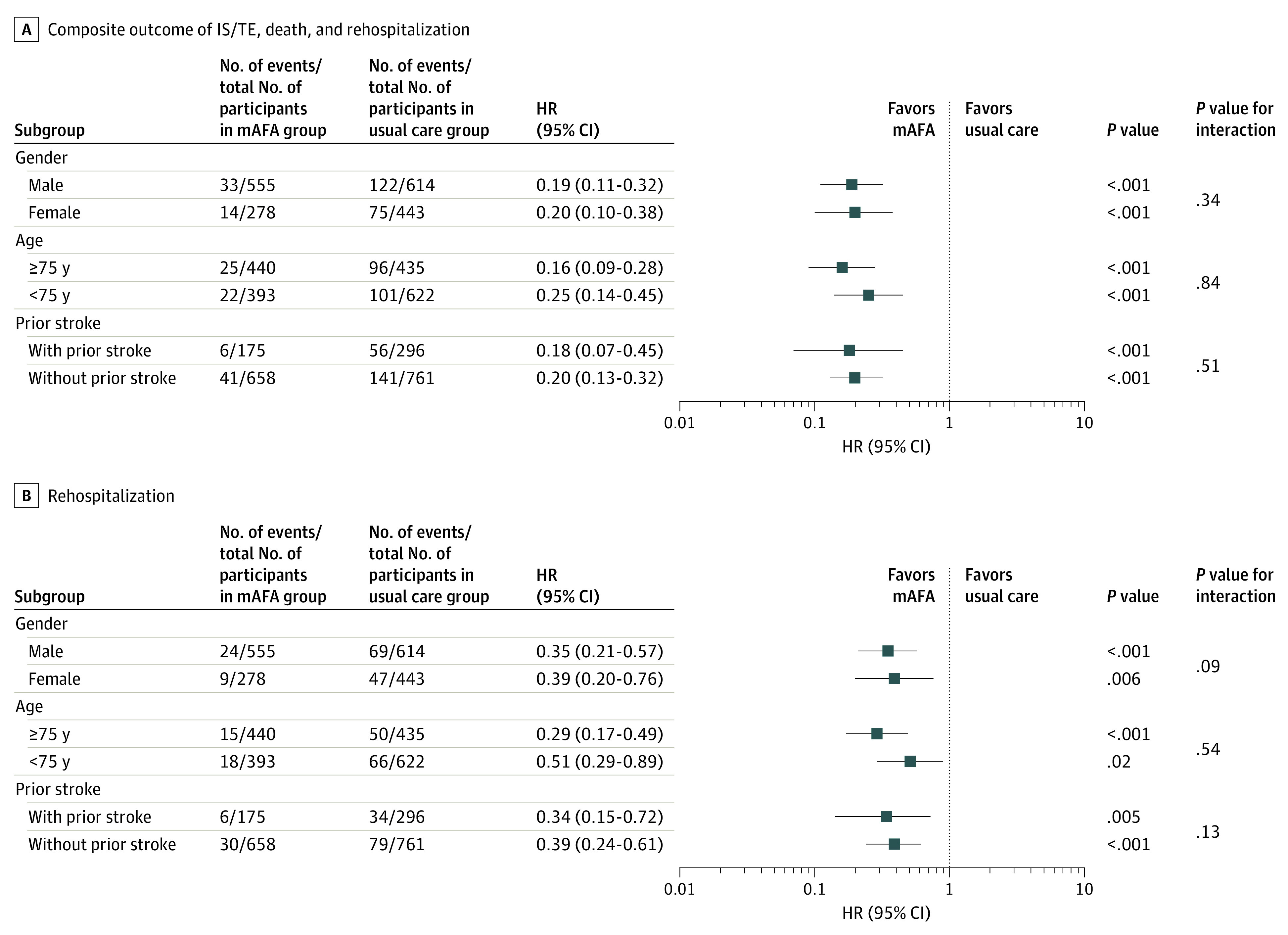
Hazard Rates of Primary Composite Outcome of Ischemic Stroke/Thromboembolism (IS/TE), Death, and Rehospitalization and Secondary End Point of Rehospitalization by Sex, Age, and Prior Stroke, Adjusting for Cluster Effect and Baseline Risk Factors There were 721 women and 1169 men; 875 patients aged 75 years or older and 1015 younger than 75 years; and 471 with prior stroke and 1419 without prior stroke. HR indicates hazard ratio; and mAFA, mobile atrial fibrillation application.

Subgroup analyses by age, prior stroke, and sex demonstrated consistently lower HRs for the primary composite outcome and rehospitalization for patients allocated to the mAFA intervention vs patients receiving usual care (Figure 3). The mAFA intervention reduced the risk of the primary composite of stroke or thromboembolism, all-cause death, and rehospitalization in patients aged 75 years or older (HR, 0.16; 95% CI, 0.09-0.28; *P* < .001) and those younger than 75 years (HR, 0.25; 95% CI, 0.14-0.45; *P* < .001) (*P* for interaction = .84) as well as in patients with prior stroke (HR, 0.18; 95% CI, 0.07-0.45; *P* < .001) and without prior stoke (HR, 0.20; 95% CI, 0.13-0.32; *P* < .001) (*P* for interaction = .51) (Figure 3A). The risk of rehospitalization was also consistently reduced in those aged 75 years or older (HR, 0.29; 95% CI, 0.17-0.49; *P* < .001) and younger than 75 years (HR, 0.51; 95% CI, 0.29-0.89; *P* = .02) (*P* for interaction = .54) as well as in patients with prior stroke (HR, 0.34; 95% CI, 0.15-0.72; *P* = .005) and without prior stoke (HR, 0.39; 95% CI, 0.24-0.61; *P* < .001) (*P* for interaction = .13) (Figure 3B).

## Discussion

In this prespecified ancillary analysis from the mAFA II trial, an mHealth technology–based integrated care approach that facilitated the implementation of the ABC pathway reduced clinical adverse events in older patients with AF and multimorbidity compared with usual care. Second, with the implementation of the A criterion, there was a reduction in thromboembolism without increasing bleeding risk, while implementation of the C criterion effectively decreased the occurrence of acute coronary syndrome, HF, and uncontrolled blood pressure vs usual care. Third, the integrated care approach of the ABC pathway in patients with multimorbidity was consistent, irrespective of age, sex, and prior stroke.

Current medical practice has tended to focus on individual conditions or disease states. However, in reality, many comorbidities do not occur in isolation but tend to cluster together.^6^ This has major implications for risks associated with AF. Health care professionals are increasingly aware of the clinical relevance of multimorbidity, but major challenges remain between health care professionals’ need for supportive tools and care pathways and stakeholders’ limited understanding of these issues.^13^ There are also challenges with designing clinical guidelines for managing multimorbidity, requiring a shift from a disease-specific approach to a more patient-centered, holistic perspective.

The high clinical risks of multimorbidity are evident from various studies, including those with AF.^5,14,15,16,17,18^ For example, in an analysis of adjudicated outcomes in patients with AF and multimorbidity enrolled in the AFFIRM trial, after a median (IQR) 3.63 (2.73-4.54) years of follow-up, the rate of the composite outcome of all-cause hospitalization and all-cause death was 37.8 per 100 patient-years^2^; hospitalization events occurred in 36.2 per 100 patient-years, all-cause death in 4.21 per 100 patient-years, and cardiovascular events in 20.3 per 100 patient-years. Nonetheless, the value of interventions to improve hard clinical outcomes in patients with AF and multimorbidity are limited. In 2018, the largest pragmatic randomized clinical trial on the management of patients with multimorbidity (1546 adults with ≥3 chronic conditions, not necessarily AF) tested the efficacy of a patient-centered approach based on several physical health dimensions, depression, and drugs/medication—a so-called 3D approach—in 33 primary care practices in the United Kingdom. The study showed no significant improvement in patients’ quality of life, the primary outcome.^19^ Alternative strategies are therefore needed.

A systematic review and meta-analysis of the use of the ABC pathway found a lower risk of all-cause death (odds ratio [OR], 0.42; 95% CI, 0.31-0.56), cardiovascular death (OR, 0.37; 95% CI, 0.23-0.58), stroke (OR, 0.55; 95% CI, 0.37-0.82), and major bleeding (OR, 0.69; 95% CI, 0.51-0.94), with management adherent to the ABC pathway compared with nonadherent management.^20^ The results were consistent in different cohorts globally.^21,22,23,24,25^ The ABC pathway has also been tested in a prospective cluster randomized trial (mAFA-II),^8^ which showed that patients allocated to the ABC pathway intervention (using mHealth technology) had lower rates of the composite outcome of ischemic stroke or systemic thromboembolism, death, and rehospitalization compared with usual care (1.9% vs 6.0%; HR, 0.39; 95% CI, 0.22 to 0.67; *P* < .001). Rates of rehospitalization were lower in the intervention group than the usual care group (1.2% vs 4.5%; HR, 0.32; 95% CI, 0.17 to 0.60; *P* < .001). In the main trial, the ABC pathway intervention also led to reduced major bleeding events and increased oral anticoagulation uptake vs usual care.^26^ The long-term extension cohort of mAFA-II showed that the beneficial impact of ABC pathway on clinical outcomes was maintained for more than 1 year with high adherence (>70%) and persistence (>90%) of the intervention.^27^ The present ancillary analysis from the mAFA-II trial found results consistent with the main trial outcomes for this high-risk cohort of older patients with AF and multimorbidity.

### Limitations

This study has limitations. It is a post hoc ancillary analysis of a clinical trial, which may not be adequately powered for some of the individual outcomes. The trial was also based on a cluster design, resulting in some imbalance with regard to clinical factors and the proportions of patients with multimorbidity in the mAFA intervention and usual care groups; however, our statistical methods have adjusted for the cluster effect.

## Conclusions

In this prespecified ancillary analysis from the mAFA-II trial, an mHealth technology–based integrated care approach that facilitates the implementation of the ABC pathway reduced meaningful clinical adverse events in older patients with AF and multimorbidity compared with usual care.

## Notes

Supplement 1.
Trial Protocol

Supplement 2. **eTable. **Treatments in mAFA Intervention and Usual Care Group

Supplement 3.
Nonauthor Collaborators

Supplement 4.
Data Sharing Statement
