# Supplementary material for: The Effects of Implementing a Mobile Health–Technology Supported Pathway on Atrial Fibrillation–Related Adverse Events Among Patients With Multimorbidity: The mAFA-II Randomized Clinical Trial
Source: JAMA Netw Open. 2021 Dec 21;4(12):e2140071. doi: 10.1001/jamanetworkopen.2021.40071 (PMC8693229; doi:10.1001/jamanetworkopen.2021.40071)
Supplement: Supplement 1. — Trial Protocol [file jamanetwopen-e2140071-s001.pdf]

SUPPLEMENTARY APPENDIX

Mobile Health (mHealth) technology for improved screening, patient involvment and optimizing integrated care in atrial fibrillation: protocol for The mAFA (mAF-App) II randomized trial

Introduction

Atrial fibrillation (AF) is the most common cardiac arrhythmia globally, with an increased risk of mortality and morbidity from stroke and heart failure [1]. Given the increasing prevalence and incidence of AF with an ageing population, this arrhythmia represents an increasing public health burden. There will be 12.1 to 15.9 million patients suffer AF in the United States by 2050, and 17.9 million people in Europe by 2060 [2,3].

Given the largest population of developing countries and the increasingly ageing population, the burden of AF is greatly increasing in China. Indeed, there has been a 20-fold increase in AF prevalence and 13-fold rise in AF-related stroke during the last 11 years in China [4]. We have previously modelling projections for the risks related with AF in East Asia, and the burden of ischaemic stroke and death remains large, despite the introduction of new guidelines and therapies [5].

An integrated, structured approach to AF care has been proposed in the 2016 European Society of Cardiology (ESC) guidelines on AF management [6], which is consistent with the Innovative Care for Chronic Conditions Framework proposal put forward by the World Health Organization [7]. Integrated AF care combines patient involvement, multidisciplinary teams, and technology tools to achieve all treatment options for AF, for example, a structured support for lifestyle changes, anticoagulation, rate control, antiarrhythmic drugs, and catheter and surgical interventions [6]. The use of the integrated care approach in AF has been associated with reduced cardiovascular hospitalisations and all-cause mortality, but not AF-related hospitalisations or cerebrovascular events [8].

Various comprehensive and complex proposals for integrated care AF management have been proposed [9]. This holistic approach to AF management may be simplified into a practical, simple ABC pathway (Avoid stroke; Better symptom management; Cardiovascular

and other comorbidity risk reduction) [10]. A major challenge is how to operationalize the concept of integrated AF care in busy "real-world" clinical practice, especially in low-middle income countries.

The individual components of the ABC (Atrial fibrillation Better Care) Pathway are facets of a holistic approach to AF patient management [10]. For example, stroke prevention with oral anticoagulation (OAC) is the cornerstone of AF management [11]. Nonetheless, suboptimal thromboprophylaxis in AF patients is highly prevalent in Asian countries [12, 13,14，15], despite the various guidelines on AF management [6,16,17,18]. Even moving into the new era of the non-vitamin K antagonist oral anticoagulants (NOACs), many patients remain undertreated in Asia [19].

Nonadherence to AF management guidelines is also common, ranging from 33% to 68% in the Middle East/Africa and Asia, respectively) [20]. Nevertheless, patient's preferences are another important reason for non-adherence of therapy [21]. Indeed, optimal stroke prevention relies on patients taking medications properly and continuously. The patient with good knowledge of AF are likely to be more concerned about a stroke and would want to be involved in joint decision-making in anticoagulant treatment [22], thus increasing treatment adherence and persistence. Lifestyle changes are also part of this comprehensive approach, whereby health lifestyle and addressing cardiovascular risk factors may improve uptake of treatments and ultimately, patient outcomes [23].

In low-middle income countries, there is a serious imbalance in the distribution of medical resources (hospitals, medical staff), for example, in China due to the unbalanced development of healthcare resources and distribution of the economy. Patients are usually admitted to the hospitals in large and medium-sized cities, but would have lost the continuing high-quality care or follow-up when they are discharged. Their medical records often cannot move with them to assist their local medical care, while the patients often do not know how to manage

themselves. Thus, a simple and cost-effective approach to AF care streamlining management pathways from the hospital to the home is still needed [24].

Besides, up to two-thirds of AF patients are asymptomatic, who are at the same risks as symptomatic AF. Detection of these AF episodes and appropriate management, will reduce the risks of AF-related stroke and other complications [25].

Novel strategies that incorporate eHealth or mobile Health (mHealth) encompasses the use of information and communication technologies in the management of disease, providing innovative solutions to the problem of long-term management after discharge [26,27]. Mobile communication and internet service are well established in China, and the high penetration rate of mobile devices and communication networks provides an excellent foundation and great opportunities for mHealth development, which may facilitate the management of AF in China [28].

In a pilot study, we designed a mHealth technology-supported AF management model (mAF App), integrating clinical decision support tools as part of patient clinical decision support tools (CHA_2DS_2-VASc, HAS-BLED, SAMe-TT_2R_2 scores), guideline-based treatment, educational materials and patient involvement strategies with self-care protocols and structured follow-up [28]. In this first prospective randomized trial of mHealth technology in patients with AF, the mAFA (mAF-App I) trial, use of the mAF App significantly improved knowledge, drug adherence, quality of life (QoL) and anticoagulation satisfaction [28].

The pilot mAFA I trial focused efforts on improving stroke prevention. Apart from stroke prevention, symptom management and lifestyle changes, as well as management of cardiovascular and other comorbidities are required in the content of AF care. Hence, we updated the mAFA platform (MAFA II) to include AF screening and integrated care for AF

using the ABC pathway, thus providing the opportunity for implementing holistic AF integrated management.

In the mAFA II trial, we hypothesized that implementation of a mHealth technology-supported AF screening and integrated ABC strategy could reduce AF-related adverse events (stroke/thromboembolism, all-cause death, and rehospitalization). Ancillary analyses would determine patient-related outcome measures, health economics and cost effectiveness, as well as an embedded qualitative study.

Study design

The mAFA II trial will be a prospective, cluster randomized controlled trial, conducted in approximately 40 centers and patients will be followed up for one year.

Study population

Inclusion criteria would include i) patients aged ≥ 18 years old, diagnosed with new-onset, paroxysmal, persistent or permanent AF confirmed with electrocardiogram (ECG) or 24 hour Holter monitors; and ii) CHA_2DS_2-VASc score ≥ 2. We excluded individuals aged <18 years old, those with mechanical prosthetic valve or moderate/severe mitral stenosis, unable to provide informed consent, or unable to have one year of follow-up for any reason.

Randomization

The 40 participating cluster hospitals will be randomized in a 1:1 ratio to receive either the mAFA intervention or usual care. A pilot feasibility investigation has been carried out for possible study sites, with respect to hospitals (size, the volume of patients for the study per month), doctors (willingness to involve in mAFA II, what are the concerns and obstacles on AF management, the possible time doctors would like to spend on patients out of discharge, study fees, etc), patient catchment (smart phone use, education level, etc.). Then the sites will be matched based on hospital size and the proportion of enrolled patients.

The hospital size is classified as big hospitals with enrollment of over 20 patients per month, and small hospitals with enrollment of under 20 patients per month, respectively. Our pilot investigation demonstrated that the ratio of big versus small hospitals was 1:2, then 142 patients for big hospitals and 71 patients for small hospitals will be needed with 10% loss of follow-up, respectively.

Intervention

All hospitals will participate in AF screening plan before suitable patients are included into the mAFA II trial. Hospitals randomsied to the intervention arm will use the mAFA platform to manage AF patients. The mAFA platform provides clinical decision support tools (CHA_2DS_2-VASc, HAS-BLED, SAMe-TT_2R_2 scores), guideline-based treatment recommendations, educational materials and patient involvement strategies with self-care protocols and structured follow-up, to support implementation of the ABC pathway for integrated or holistic AF management.

Avoid stroke

OACs management will include personalized OACs management tailored to the patients. For example, the time in therapeutically range (TTR) will be automatically calculated for the patients on warfarin. If the patients were take dabigatran or rivaroxaban (the only 2 NOACs that are approved in China), the relative methods of taking drug and the things needing to be attended, will be provided by mAFA. Drug adherence will be recorded, as patients can use mAFA to record their dose and drug use.

Liver or renal function monitoring plan will be recommended to the patients matched the patient's age, comorbidities, co-medicine, and the use of OACs. Modifiable bleeding risk factors would be flagged up and addressed in all patients, and bleeding risk strata would be dynamically assessed using the HAS-BLED score.

Patient-reported thromboembolism or bleeding events would be captured using the structured questionnaire developed by the mAFA platform. Doctors can also communicate with patients on these events through instant message on mAFA.

Better symptom management

Classification and assessment of symptom will include use of the European Heart Rhythm Association (EHRA) AF symptom assessment. Any chest tightness will be categorized using the Canadian Cardiovascular Society Angina Classification. Tiredness and exercise capacity will be recorded based on the New York Heart Assessment (NYHA) functional assessment. In paroxysmal AF patients, cardiac rhythm monitoring will be undertaken with PhotoPlethysmo Graphy technology (Preventicus GmbH, Jena, Germany).

Cardiovascular and other comorbidities management

This will include lifestyle recommendations including, the matched healthy diet based on the patient's comorbidities and other cardiovascular risk (e.g. low-salt diet for hypertension, low-fat diet for hyperlipemia, etc.), regular exercise (e.g. at least 30 min/day,5 days/week of moderate intensity physical activity, etc.), weight reduction, smoking cessation, etc. Blood pressures will be recorded, and suboptimal readings would be 'flagged up' for optimization of treatment. Heart failure and angina management would be optimized, as needed. Pulse Oxygen Saturation (SpO2) will be monitored and those with suspected sleep apnea will be flagged up for formal assessment and management.

Follow up and study outcomes

All patients will be followed in the outpatient clinics at 6 and 12 months for clinical events. The clinical events will be adjudicated by Clinical events committee.

The *primary endpoint* is the composite of stroke/thromboembolism, all-cause death, and rehospitalization. Thromboembolism endpoint includes ischaemic stroke, transient ischemic attack (TIA), pulmonary embolism, deep vein thromboembolism (DVT), other thromboembolism (peripheral embolism, atrial thrombus and left atrial appendage thrombus, etc.). All-cause death will include cardiac death, vascular death, and non-cardiovascular death. Cardiac death includes death caused by ST-segment elevation myocardial infarction /Non-ST-segment elevation myocardial infarction (STEMI/NSTEMI), heart failure (HF), arrhythmia, cardiac perforation / tamponade, and other deaths of cardiac origin. Vascular death will include death ascribed to ischemic stroke, haemorrhagic stroke, systemic haemorrhage, peripheral embolism, and pulmonary embolism. Rehospitalization for AF and AF-related complications, will include stroke, systemic thromboembolism, angina, STEMI/NSTEMI, HF, etc.

Secondary outcomes will include the following: i) the change in proportion of patients able to continue anticoagulation; ii) the mAFA intervention costs, individual-level HealthCare Resource Utilization (HCRU) as well as associated costs, and quality adjusted life year (QALY) gained with mAFA use compared to usual care; and iii) event rates: event rate for composite of ischaemic stroke/TIA and systemic thromboembolism, HF, cardiovascular death, or rehospitalization for any cause for AF.

mAFA training, data management, monitoring and quality control

The mAFA trial program will deliver the training on mAF App use for the researchers before the study. Self-reported healthcare utilisation including medicine use, visits for AF-related adverse outcomes, hospitalisations, etc. will be assessed by AF cost questionnaires at 6 and 12 months. Patients will also be asked to fill in a patient-specific cost diary every month during study period to avoid missing information.

An independent third party (CheckTruth, Ltd, Beijing, China) will monitor the project onsite, ensuring health, safety and the relevant rights of subjects are protected. Monitoring will also ensure the sites carry out the study according to protocol, the data collected are true and accurate, and the site staff and facility meet the protocol requirements.

All sites that enroll at least one patient will undergo a data control audit by completing a site visit. The visits will spread over the entire study period, with first visit of approximately 30% being done around the time of enrollment. In addition, all sites would undergo further data monitoring as necessary, based on performance, queries initiated or missing data. For the sites undergoing monitoring, the case report forms for patients enrolled at site will be monitored for source documentation and accuracy.

Statistical analysis

Analyses will be conducted according to the intention-to-treat principle [29]. The primary analyses of primary, secondary and exploratory outcomes will be based on the intention-to-treat population adjusted for the effect of clustering. All primary tests of significance will be two-sided with alpha=5%. Frequencies and percentages per group as well as hazard ratios with 95% confidence interval (CI) will be reported for binary outcomes. Continuous variables and rate variables will be summarised using mean, standard deviation, 25, 50 and 75 percentiles, and minimum and maximum values. All statistical analyses will be completed with IBM SPSS Statistics, version 22.0 (SPSS Inc)

Cox proportional hazard model analysis will be used to assess the effect of mAFA intervention on the primary composite outcome of stroke/thromboembolism, all-cause death, and rehospitalization. Additionally, the impact of the mAFA intervention on clinical outcomes will be explored, including the time to first occurrence of ischaemic stroke/TIA and systemic thromboembolism, rehospitalization, and cardiovascular death will be analyzed, in relation to age, sex, multimorbidity, etc. Change in proportion of patients on

anticoauglation will be evaluated with Mantel-Haenszel statistics as adjusted for the effect of clustering.

Subgroup and sensitivity analyses

The subgroup analyses for the primary and secondary outcomes will be conducted by age strata, gender, and educational level. Sensitivity analyses of the primary and secondary outcomes will be repeated among all randomized patients without major protocol violations and classified according to the intervention to which they were randomized.

Health economic evaluation

The healthcare resource utilization (hospitalizations, physician office visits, etc.) and healthcare costs (hospitalizations, primary care, medications, etc.) of mAFA and usual care will be examined by descriptive statistics.

For mAFA intervention costs, the included costs are those that are likely to differ across the mAFA intervention and usual care, specifically the costs of:

(i) Costs of the mAF app design (for patients and doctors) and also associated Apps including AF decision support tool, and other apps to provide stroke and bleeding risk calculations. These costs can be derived from financial statement or unit costs multiplied by total personnel-time.

(ii) Costs resulting from additional time spent by patients in learning the App, uploading their laboratory tests, learning educational programs, and their involvement with self-care, etc.

(iii) Costs of the app-integrated Patient's Educational Program development.

(iv) Personnel costs spent in double check the structured data and the source documentation of mAF App.

(v) Costs resulting from additional time spent by doctors compared to control group.

Protocol-driven costs for research purposes shall not be included, which may also be balanced between arms.

MAFA intervention differences in the mean number of health care resources utilized and in the average rate per unit of time will be estimated. Further, the longitudinal models will be utilized for the analysis of health care resource use data [30].

The Kaplan–Meier method will be used for the analysis of cost data, considering the presence of censoring in the clinical trial data [31].

The cost-effectiveness (improvements in life years, quality of life, quality-adjusted life years (QALYs), the cost per QALY gained, and the incremental cost-effectiveness ratio (ICER)) of mAFA compared to usual care will be calculated. The economic assessments will be reported in alignment with the Consolidated Health Economic Evaluation Reporting Standards (CHEERS) statement [32]. The bootstrap method will be used to construct the confidence interval for the ICER [33].

Power calculation

An intracluster correlation coefficient (ICC) is assumed to be 0.02, and a reduction in stroke risk would be 52% after intervention (Hazard ratio, HR 0.48, 95% CI 0.23-0.99) according to a prior study, IMPACT-AF [34]. There would be 10% difference of anticoagulant uptake after mAFA intervention and after usual care [28], so we assume baseline anticoagulant use of 40%, with a post-mAFA intervention use of 60% and a post-control arm rate of 50%, respectively. The composite adverse events (stroke/thromboembolism, all-cause death, and rehospitalization) is assumed as 10% during first one year with baseline anticoagulant use of 40%. The sample size will be 3294 patients with type I error under 5% and power over 90%. Considered 10% loss to follow-up, a total of 3660 patients will be needed, which randomized into MAFA arm and usual care arm, respectively [35].

Discussion

The increasing global burden of AF leads to a high incidence of stroke, systemic embolism, heart failure, and death. In recent years, new anticoagulant drugs (NOACs) and technologies (cryoballoon ablation, percutaneous left atrial appendage occlusion, etc) have been introduced for the treatment of patients with AF. Despite this, suboptimal management is common, and all-cause or cardiovascular death remains high amongst the AF population [36]. Indeed, death is the most frequent adverse event in AF, with CHF, MI, stroke and major bleeding contributing to AF mortality [37,38].

The comorbidities associated with the worse outcomes in AF patients are often sub-optimally treated with guideline-recommended drugs. Thus, AF integrated care has been proposed to provide a holistic approach to AF management and improve outcome. Integrated AF care requires patient involvement and empowerment, lifestyle changes, educational guidance and shared decision-making. Use of new technologies may facilitate this, especially in healthcare systems with a high penetration rate of mobile devices and communication networks.

This was tested in the pilot mAFA I trial, which was a small study which showed that mHealth technology-supported AF management strategy was feasible, effective and safe [28]. The clinical decision support provided by the mAF App streamlined guideline-based decision-making for the stroke prevention in patients with AF, and was easily handled by doctors and understood by patients. The clinical decision support tools in the mAF App automatically assessed stroke and bleeding risk, stratified the patients at high-risk of stroke/TE to anticoagulant treatment, while balancing bleeding risks. Bleeding risk factors were also labeled, and could be reviewed by doctors and patients for correction of the modifiable risk factors. Personalized choice of OAC could also be advised based on the SAMe-TT_2R_2 score, resulting in rational decision-making on anticoagulant management options, with patient engagement.

The mAF App then automatically made a follow-up plan, permitting patient's self-monitoring and timely feedback. Indeed, the pilot mAFA study (mAFA I trial) also showed that the mAF App-based self-monitoring and feedback enhanced compliance and adherence of drug therapy and anti-coagulant satisfaction [28].

The mAFA I study focused on stroke/TE prevention, but it is well-recognised that interventions beyond anticoagulation are needed to further reduce mortality in AF. Thus, the objective of the preseny study (mAFA II) is to develop and implement a holistic approach to integrated AF management, covering AF screening, prevention strategies (oral anticoagulation, symptom management with rate or rhythm control), and risk factor management with the aim of reducing recurrent stroke, HF, rehospitalization, and death, etc.

Screening strategies can improve the detection of AF in high-risk population [39], and could modify morbidity and mortality by early institution of preventive therapies, such as OAC. Thus, a cost-effective, screening strategy using PhotoPlethysmo Graphy(PPG) technology has been integrated into the mAFA II study to provide better AF care, balancing the correct detection tool with the targeted at-risk population [40].

Conclusion

The mAFA II trial will provide evidence for an integrated care approach to holistic AF care, supported by mobile health technology to improve screening, patient involvment and optimizing management. This trial tests an innovative solution to reduce AF-related stroke/systemic thromboembolism, all-cause death, with patient involvement and empowerment, educational guidance and shared decision-making.

References

1.Chugh SS, Havmoeller R, Narayanan K, Singh D, Rienstra M, Benjamin EJ, Gillum RF, Kim YH, McAnulty JH Jr, Zheng ZJ, Forouzanfar MH, Naghavi M, Mensah GA, Ezzati M, Murray CJ. Worldwide epidemiology of atrial fibrillation: a Global Burden of Disease 2010 Study. Circulation. 2014;129(8):837-847.

2.Krijthe BP, Kunst A, Benjamin EJ, Lip GY, Franco OH, Hofman A, Witteman JC, Stricker BH, Heeringa J. Projections on the number of individuals with atrial fibrillation in the European Union, from 2000 to 2060. Eur Heart J. 2013;34:2746–2751.

3.Miyasaka Y, Barnes ME, Gersh BJ, Cha SS, Bailey KR, Abhayaratna WP, Seward JB, Tsang TS. Secular trends in incidence of atrial fibrillation in Olmsted County, Minnesota, 1980 to 2000, and implications on the projections for future prevalence. Circulation. 2006;114:119–125.

4. Guo Y, Tian Y, Wang H, Si Q, Wang Y, Lip GY. Prevalence, incidence, and lifetime risk of atrial fibrillation in China: new insights into the global burden of atrial fibrillation. Chest. 2015;147(1):109-19.

5. Bai Y, Guo SD, Shantsila A, Lip GYH. Modelling projections for the risks related with atrial fibrillation in East Asia: a focus on ischaemic stroke and death. Europace. 2017 Nov 20. doi: 10.1093/europace/eux328.

6.Kirchhof P, Benussi S, Kotecha D, Ahlsson A, Atar D, Casadei B, Castella M, Diener HC, Heidbuchel H, Hendriks J, Hindricks G, Manolis AS, Oldgren J, Popescu BA, Schotten U, Van Putte B, Vardas P, Agewall S, Camm J, Baron Esquivias G, Budts W, Carerj S, Casselman F, Coca A, De Caterina R, Deftereos S, Dobrev D, Ferro JM, Filippatos G, Fitzsimons D, Gorenek B, Guenoun M, Hohnloser SH, Kolh P, Lip GY, Manolis A, McMurray J, Ponikowski P, Rosenhek R, Ruschitzka F, Savelieva I, Sharma S, Suwalski P, Tamargo JL, Taylor CJ, Van Gelder IC, Voors AA, Windecker S, Zamorano JL, Zeppenfeld K. 2016 ESC Guidelines for the management of atrial fibrillation developed in collaboration with EACTS. Eur Heart J. 2016; 37: 2893-2962.

7.Nuno R, Coleman K, Bengoa R, Sauto R. Integrated care for chronic conditions: the contribution of the ICCC Framework. Health Policy. 2012; 105:55–64.

8. Gallagher C, Elliott AD, Wong CX, Rangnekar G, Middeldorp ME, Mahajan R, Lau DH, Sanders P, Hendriks JML. Integrated care in atrial fibrillation: a systematic review and meta-analysis. Heart. 2017;103(24):1947-1953.

9.Kirchhof P. The future of atrial fibrillation management: integrated care and stratified therapy. Lancet 2017 Apr 28. doi: 10.1016/S0140-6736(17)31072-3.

10. Lip GYH. The ABC pathway: an integrated approach to improve AF management. Nat Rev Cardiol. 2017;14(11):627-628.

11. Lip G, Freedman B, De Caterina R, Potpara TS.Stroke prevention in atrial fibrillation: Past, present and future. Comparing the guidelines and practical decision-making. Thromb Haemost. 2017;117(7):1230-1239.

12.Lip GYH, Brechin CM, Lane DA. The global burden of atrial fibrillation and stroke: a systematic review of the epidemiology of atrial fibrillation in regions outside North America and Europe. Chest. 2012;142(6):1489-1498.

13.Guo Y, Wang H, Tian Y, et al. Time Trends of Aspirin and Warfarin Use on Stroke and Bleeding Events in Chinese Patients With New-Onset Atrial Fibrillation. Chest. 2015; 148: 62-72.

14.Guo Y, Pisters R, Apostolakis S, Blann AD, Wang H, Zhao X, Zhang Y, Zhang D, Ma J, Wang Y, Lip GY. Stroke risk and suboptimal thromboprophylaxis in Chinese patients with atrial fibrillation: would the novel oral anticoagulants have an impact? Int J Cardiol. 2013;168(1):515-522.

15. Bai Y, Wang YL, Shantsila A, Lip GYH.The Global Burden of Atrial Fibrillation and Stroke: A Systematic Review of the Clinical Epidemiology of Atrial Fibrillation in Asia. Chest. 2017;152(4):810-820.

16.Chiang CE, Wu TJ, Ueng KC, et al. 2016 Guidelines of the Taiwan Heart Rhythm Society and the Taiwan Society of Cardiology for the management of atrial fibrillation. Journal of the Formosan Medical Association = Taiwan yi zhi. 2016;115(11):893-952.

17.Writing Committee for Expert Consensus on the Management of Atrial Fibrillation in Elderly Population, Chinese Geriatric Society, Editorial Board of Chinese Journal of Geriatrics. Expert consensus on the management of atrial fibrillation in elderly population (2016). Chin J Geriatr. 2016,35(09): 915-928. Available at: http://zhlnyxzz.yiigle.com/CN112225201609/930117.jhtml. Accessed: March 2018.

18.Chiang CE, Okumura K, Zhang S, et al. 2017 consensus of the Asia Pacific Heart Rhythm Society on stroke prevention in atrial fibrillation. J Arrhythm. 2017;33(4):345-367.

19. Mazurek M, Huisman MV, Rothman KJ, Paquette M, Teutsch C, Diener HC, Dubner SJ, Halperin JL, Ma CS, Zint K, Elsaesser A, Lu S, Lip GYH; GLORIA-AF Investigators.Regional Differences in Antithrombotic Treatment for Atrial Fibrillation: Insights from the GLORIA-AF Phase II Registry. Thromb Haemost. 2017;117(12):2376-2388.

20.Gamra H, Murin J, Chiang C-E, et al. Use of antithrombotics in atrial fibrillation in Africa, Europe, Asia and South America: Insights from the International RealiseAF Survey. Arch Cardiovasc Dis. 2014; 107: 77–87.

21.Raparelli V, Proietti M, Cangemi R, et al. Adherence to oral anticoagulant therapy in patients with atrial fibrillation. Focus on non-vitamin K antagonist oral anticoagulants. Thromb Haemost. 2017; 117: 209-218.

22.Clarkesmith DE, Lip GYH, Lane DA. Patients' experiences of atrial fibrillation and non-vitamin K antagonist oral anticoagulants (NOACs), and their educational needs: a qualitative study. Thromb Res. 2017; 153:19-27.

23. Lau DH, Nattel S, Kalman JM, Sanders P.Modifiable Risk Factors and Atrial Fibrillation. Circulation. 2017;136(6):583-596.

24. Sun J, Guo Y, Wang X, Zeng Q. mHealth For Aging China: Opportunities and Challenges. Aging Dis. 2016;7(1):53-67.

25. Freedman B, Camm J, Calkins H, Healey JS, Rosenqvist M, Wang J, Albert CM, Anderson CS, Antoniou S, Benjamin EJ, Boriani G, Brachmann J, Brandes A, Chao TF, Conen D, Engdahl J, Fauchier L, Fitzmaurice DA, Friberg L, Gersh BJ, Gladstone DJ, Glotzer TV, Gwynne K, Hankey GJ, Harbison J, Hillis GS, Hills MT, Kamel H, Kirchhof P, Kowey PR, Krieger D, Lee VWY, Levin LÅ, Lip GYH, Lobban T, Lowres N, Mairesse GH, Martinez C, Neubeck L, Orchard J, Piccini JP, Poppe K, Potpara TS, Puererfellner H, Rienstra M, Sandhu RK, Schnabel RB, Siu CW, Steinhubl S, Svendsen JH, Svennberg E, Themistoclakis S, Tieleman RG, Turakhia MP, Tveit A, Uittenbogaart SB, Van Gelder IC, Verma A, Wachter R, Yan BP; AF-Screen Collaborators.Screening for Atrial

Fibrillation: A Report of the AF-SCREEN International Collaboration. Circulation. 2017;135(19):1851-1867.

26.Burke LE, Ma J, Azar KM, et al; American Heart Association Publications Committee of the Council on Epidemiology and Prevention, Behavior Change Committee of the Council on Cardiometabolic Health, Council on Cardiovascular and Stroke Nursing, Council on Functional Genomics and Translational Biology, Council on Quality of Care and Outcomes Research, and Stroke Council. Current Science on Consumer Use of Mobile Health for Cardiovascular Disease Prevention: A Scientific Statement From the American Heart Association. Circulation. 2015; 132: 1157-213.

27.Cowie MR, Bax J, Bruining N, et al. e-Health: a position statement of the European Society of Cardiology. Eur Heart J. 2016; 37: 63-66.

28.Guo Y, Chen Y, Lane DA,Liu L,Wang Y, Lip GYH. Mobile Health (mHealth) technology integrating clinical decision support and patient involvement for the management of patients with atrial fibrillation: The mAFA (mAF-App) randomized trial. Am J Medicine 2017.http://http://dx.doi.org/10.1016/j.amjmed.201 7.07.003.

29. Fergusson D, Aaron SD, Guyatt G, Hebert P. Post-randomisation exclusions: the intention to treat principle and excluding patients from analysis. BMJ. 2002;325(7365):652–654.

30. Itzler R, Dasbach E, Koch G, Heyse JF. Using longitudinal models for the analysis of resource use data: the case of asthma. Economic assessment in clinical trials. Drug Information Association Meeting, Orlando, Florida, November 1999.

31. Fenn P, McGuire A, Phillips V, Backhouse M, Jones D. The analysis of censored treatment cost data in economic evaluation. Medical Care 1995; 33: 851–863.

32. Husereau D, Drummond M, Petrou S, Carswell C, Moher D, Greenberg D, Augustovski F, Briggs AH, Mauskopf J, Loder E. Consolidated Health Economic Evaluation Reporting Standards (CHEERS)-explanation and elaboration: a report of the ISPOR Health Economic Evaluation Publication Guidelines Good Reporting Practices Task Force. Value Health. 2013;16(2):231–250.

33. Chen S, Zhao H.Estimating incremental cost-effectiveness ratios and their confidence intervals with different terminating events for survival time and costs.Biostatistics. 2013 Jul;14(3):422-432.

34. Vinereanu D, Lopes RD, Bahit MC, Xavier D, Jiang J, Al-Khalidi HR, He W, Xian Y, Ciobanu AO, Kamath DY, Fox KA, Rao MP, Pokorney SD, Berwanger O, Tajer C, de Barros E Silva PGM, Roettig ML, Huo Y, Granger CB; IMPACT-AF investigators. A multifaceted intervention to improve treatment with oral anticoagulants in atrial fibrillation (IMPACT-AF): an international, cluster-randomised trial. Lancet. 2017;390(10104):1737-1746.

35. Eldridge SM, Ashby D, Kerry S. Sample size for cluster randomized trials: effect of coefficient of variation of cluster size and analysis method. Int J Epidemiol. 2006;35(5):1292-1300.

36. Proietti M, Laroche C, Opolski G, Maggioni AP, Boriani G, Lip GYH; AF Gen Pilot Investigators. 'Real-world' atrial fibrillation management in Europe: observations from the 2-year follow-up of the EURObservational Research Programme-Atrial Fibrillation General Registry Pilot Phase. Europace. 2017;19(5):722-733.

37. Piccini JP, Hammill BG, Sinner MF, Hernandez AF, Walkey AJ, Benjamin EJ, Curtis LH, Heckbert SR. Clinical course of atrial fibrillation in older adults: the importance of cardiovascular events beyond stroke. Eur Heart J. 2014;35(4):250-256.

38. Gómez-Outes A, Lagunar-Ruíz J, Terleira-Fernández AI, Calvo-Rojas G, Suárez-Gea ML, Vargas-Castrillón E. Causes of Death in Anticoagulated Patients With Atrial Fibrillation. J Am Coll Cardiol. 2016;68(23):2508-2521.

39. Krivoshei L, Weber S, Burkard T, Maseli A, Brasier N, Kühne M, Conen D, Huebner T, Seeck A, Eckstein J. Smart detection of atrial fibrillation. Europace. 2017;19(5):753-757.

40. N Brasier, CJ Raichle, et al. Detection of atrial fibrillation with a smartphone camera: First prospective, international, multicenter, clinical validation study (DETECT AF PRO). EHRA 2018 (late breaking clinical trials).
