# Supplementary material for: The Effects of Implementing a Mobile Health–Technology Supported Pathway on Atrial Fibrillation–Related Adverse Events Among Patients With Multimorbidity: The mAFA-II Randomized Clinical Trial
Source: JAMA Netw Open. 2021 Dec 21;4(12):e2140071. doi: 10.1001/jamanetworkopen.2021.40071 (PMC8693229; doi:10.1001/jamanetworkopen.2021.40071)
Supplement: Supplement 2. — eTable. Treatments in mAFA Intervention and Usual Care Group [file jamanetwopen-e2140071-s002.pdf]

Supplemental Online Content

Yao Y, Guo Y, Lip GYH; the mAF-App II Trial investigators. The effects of implementing a mobile health–technology supported pathway on atrial fibrillation–related adverse events among patients with multimorbidity: the mAFA-II randomized clinical trial. *JAMA Netw Open.* 2021;4(12):e2140071. doi:10.1001/jamanetworkopen.2021.40071

eTable. Treatments in mAFA Intervention and Usual Care Group

This supplemental material has been provided by the authors to give readers additional information about their work.

© 2021 Yao Y et al. *JAMA Network Open.*

eTable. Treatments in mAFA Intervention and Usual Care Group

	mAFA (n=833)		Usual care (n=1057)	
**Pathway A**				
Warfarin, n (%)	48	5.76%	176	16.65%
Dabigatran, n (%)	585	70.20%	205	19.40%
Rivaroxaban, n (%)	101	12.10%	264	25.00%
NOAC, n (%)	686	82.30%	469	44.40%
**Pathway B**				
Propafenone, n (%)	13	1.56%	28	2.65%
Amiodarone, n (%)	89	10.68%	50	4.73%
ß blockers, n (%)	278	33.40%	200	18.92%
**Pathway C**				
ACEI/ARB, n (%)	269	35.77%	264	24.98%
Calcium channel blockers, n (%)	162	19.45%	147	13.91%
Statins, n (%)	352	42.26%	348	32.92%

* NOAC: non-vitamin K antagonist oral anticoagulant inhibitor. ACEI: angiotensin converting enzyme inhibitors. ARB: angiotensin II receptor blocker.

© 2021 Yao Y et al. *JAMA Network Open.*
