# Supplementary material for: The Effects of Implementing a Mobile Health–Technology Supported Pathway on Atrial Fibrillation–Related Adverse Events Among Patients With Multimorbidity: The mAFA-II Randomized Clinical Trial
Source: JAMA Netw Open. 2021 Dec 21;4(12):e2140071. doi: 10.1001/jamanetworkopen.2021.40071 (PMC8693229; doi:10.1001/jamanetworkopen.2021.40071)
Supplement: Supplement 3. — Nonauthor Collaborators [file jamanetwopen-e2140071-s003.pdf]

Supplemental Online Content: Nonauthor Collaborators

*Indicates required information. Only first name, last name, and suffix will appear in PubMed.

| *Group Name(s): mAF-App II Trial investigators | | | | | | | |
|---|---|---|---|---|---|---|---|
| ***First Name and Middle Initial(s)** | ***Last Name** | ***Suffix (eg, Jr, III)** | Academic Degrees | Institution | Location (city, state/province, country) | Role or Contribution, eg, chair, principal investigator | Group (if more than 1 Group listed in the byline) and/or Subgroup (eg, Steering Committee) |
| Yutao | Guo | | Doctor | Chinese PLA General Hospital | Beijing,China | Co-Chair | Executive Steering Committee |
| Gregory Y H | Lip | | Doctor | Institute of Cardiovascular Sciences, University of Birmingham, UK; Liverpool Centre for Cardiovascular Science, University of Liverpool | Birmingham, UK | Co-Chair | Executive Steering Committee |
| Deirdre A. | Lane | | Doctor | Liverpool Centre for Cardiovascular Science, University of Liverpool | Liverpool， UK | principal investigator | Executive Steering Committee |
| Yundai | Chen | | Doctor | Chinese PLA General Hospital | Beijing, China | principal investigator | Executive Steering Committee |
| Liming | Wang | | Doctor | The National Center for Chronic and Noncommunicable Disease Control and Prevention, Chinese Center for Disease Control and Prevention | Beijing, China | principal investigator | Executive Steering Committee |
| Jens | Eckstein | | Doctor | University Hospital Basel | Switzerland | principal investigator | Steering committee |
| G Neil | Thomas | | Doctor | Institute of Applied Health Research, University of Birmingham, | Birmingham, UK | principal investigator | Steering committee |
| Tong | Liu | | Doctor | The Second Hospital of Tianjin Medical University | Tianjin,Cina | principal investigator | Steering committee |
| Mei | Feng | | Doctor | Shanxi Dayi Hospital, | Taiyuan, Shanxi, China | principal investigator | Steering committee |
| Xuejun | Liu | | Doctor | Affiliated First Hospital, Shanxi Medical University, | China | principal investigator | Steering committee |
| Xiaoming | Li | | Doctor | Cardiovascular Disease Hospital of Shanxi Province, | China | principal investigator | Steering committee |
| Zhaoliang | Shan | | Doctor | PLA General Hospital, | Beijing, China | principal investigator | Steering committee |

*Indicates required information. Only first name, last name, and suffix will appear in PubMed.

| *First Name and Middle Initial(s) | *Last Name | *Suffix (eg, Jr, III) | Academic Degrees | Institution | Location (city, state/province, country) | Role or Contribution, eg, chair, principal investigator | Group (if more than 1 Group listed in the byline) and/or Subgroup (eg, Steering Committee) |
|---|---|---|---|---|---|---|---|
| Xiangming | Shi | | Doctor | PLA General Hospital, | Beijing, China | principal investigator | Steering committee |
| Wei | Zhang | | Doctor | PLA Army General Hospital, | Beijing, China | principal investigator | Steering committee |
| Yunli | Xing | | Doctor | Beijing Friendship Hospital, Capital Medical University | Beijing, China | principal investigator | Steering committee |
| Jing | Wen | | Doctor | Beijing Haidian Hospital | Beijing, China | principal investigator | Steering committee |
| Fan | Wu | | Doctor | Tianjin Medical University General Hospital, | Tianjing, China | principal investigator | Steering committee |
| Sitong | Yang | | Doctor | The First Affiliated Hospital, Ji Lin University, | Ji Lin, China | principal investigator | Steering committee |
| Xiaoqing | Jin | | Doctor | Tongji Hospital,Tongji medical College, Huazhong University Of Science & Technology, | Wuhan, China | principal investigator | Steering committee |
| Bo | Yang | | Doctor | Xiangya Hospital Central South University, | Changsha, China | principal investigator | Steering committee |
| Xiaojuan | Bai | | Doctor | ShengJing Hospital of China Medical University, | Shengyang, China | principal investigator | Steering committee |
| Yuting | Jiang | | Doctor | Suqian Hospital, | Jiangsu, China | principal investigator | Steering committee |
| Yangxia | Liu | | Doctor | General Hospital of Shengyang Military, | Shengyang, China | principal investigator | Steering committee |
| Yingying | Song | | Doctor | Bozhou Renmin Hospital, | Anhui, China | principal investigator | Steering committee |
| Zhongju | Tan | | Doctor | The First Hospital of Zhejiang Province, | Hangzhou, China | principal investigator | Steering committee |
| Li | Yang | | Doctor | Yunnan Cardiovascular Hospital, | Kunming, China | principal investigator | Steering committee |
| Tianzhu | Luan | | Doctor | The First Affiliated Hospital of Haerbing Medical University, | Haerbing, China | principal investigator | Steering committee |
| Chunfeng | Niu | | Doctor | The Second Affiliated Hospital of Haerbing Medical University, | Haerbing, China | principal investigator | Steering committee |
| Lili | Zhang | | Doctor | The Fourth Affiliated Hospital of Haerbing Medical University, | Haerbing, China | principal investigator | Steering committee |
| Shuyan | Li | | Doctor | The First Affiliated Hospital, Ji Lin University, | Ji Lin, China | principal investigator | Steering committee |

Supplemental Online Content: Nonauthor Collaborators

*Indicates required information. Only first name, last name, and suffix will appear in PubMed.

| *First Name and Middle Initial(s) | *Last Name | *Suffix (eg, Jr, III) | Academic Degrees | Institution | Location (city, state/province, country) | Role or Contribution, eg, chair, principal investigator | Group (if more than 1 Group listed in the byline) and/or Subgroup (eg, Steering Committee) |
|---|---|---|---|---|---|---|---|
| Zulu | Wang | | Doctor | General Hospital of Shengyang Military, | Shengyang, China | principal investigator | Steering committee |
| Bing | Xv | | Doctor | The First People's Hospital of Shengyang, | Shengyang, China | principal investigator | Steering committee |
| Liming | Liu | | Doctor | The Second Afficated Hospital of Shengyang Medical University, | Shengyang, China | principal investigator | Steering committee |
| Yuanzhe | Jin | | Doctor | The Fourth Affilicated Hospital of China Medical University, | Shengyang, China | principal investigator | Steering committee |
| Yunlong | Xia | | Doctor | The First Affiliated Hospital of Dalian Medical University, | Dalian, China | principal investigator | Steering committee |
| Xiaohong | Chen | | Doctor | The People's Hospital of Liaoning Province, | Shengyang, China | principal investigator | Steering committee |
| Fang | Wu | | Doctor | Rui Jin Hospital, Tong university School of Medicine, | Shanghai, China | principal investigator | Steering committee |
| Lina | Zhong | | Doctor | The Affiliated Hospital of Qingdao University, | Qingdao, China | principal investigator | Steering committee |
| Yihong | Sun | | Doctor | China-Japan Friendship Hospital, | Beijing, China | principal investigator | Steering committee |
| Shujie | Jia | | Doctor | Beijing Anzhen Hospital, Capital Medical University, | Beijing, China | principal investigator | Steering committee |
| Jing | Li | | Doctor | Xuanwu Hospital Capital Medical University, | Beijing, China | principal investigator | Steering committee |
| Nan | Li | | Doctor | The Third People's Hospital of Dalian, Dalian,China | Dalian,China | principal investigator | Steering committee |
| Shijun | Li | | Doctor | Dalian Muncipal Central Hospital Affiliated of Dalian Medical University, | Dalian,China | principal investigator | Steering committee |
| Huixia | Liu | | Doctor | Guangdong Academy of Medical Sciences Guangdong General Hospital, | Guangdong,China | principal investigator | Steering committee |

Supplemental Online Content: Nonauthor Collaborators

*Indicates required information. Only first name, last name, and suffix will appear in PubMed.

| *First Name and Middle Initial(s) | *Last Name | *Suffix (eg, Jr, III) | Academic Degrees | Institution | Location (city, state/province, country) | Role or Contribution, eg, chair, principal investigator | Group (if more than 1 Group listed in the byline) and/or Subgroup (eg, Steering Committee) |
|---|---|---|---|---|---|---|---|
| Rong | Li | | Doctor | The First Affiliated Hospital of Guangzhou University of Traditional Chinese Medicine, | Guangzhou,China | principal investigator | Steering committee |
| Fan | Liu | | Doctor | The Second Hospital of Hebei Medical University, | Hebei,China | principal investigator | Steering committee |
| Qingfeng | Ge | | Doctor | North China University Science And Technology Affiliated Hospital | Tangshan， China | principal investigator | Steering committee |
| Tianyun | Guan | | Doctor | The Second Hospital of Jilin University, | Jilin,China | principal investigator | Steering committee |
| Yuan | Wen | | Doctor | The Second Affiliated Hospital of Nanchang University, | Nanchang,China | principal investigator | Steering committee |
| Xin | Li | | Doctor | BenQ Hospital affiliated to Nanjing Medical University, | Nanjing,China | principal investigator | Steering committee |
| Yan | Ren | | Doctor | Ruijin Hospital, Shanghai Jiao Tong University School of Medicine | Shanghai, China | principal investigator | Steering committee |
| Xiaoping | Chen | | Doctor | Taiyuan City Central Hospital, | Taiyuan,China | principal investigator | Steering committee |
| Ronghua | Chen | | Doctor | Tangshan People's Hospital, | Tangshan,China | principal investigator | Steering committee |
| Yun | Shi | | Doctor | Tianjin Fourth Central Hospital | Tianjin,China | principal investigator | Steering committee |
| Yulan | Zhao | | Doctor | The Second Affiliated Hospital of Zhengzhou University, | Zhengzhou, China | principal investigator | Steering committee |
| Haili | Shi | | Doctor | Zhengzhou Central Hospital Affiliated to Zhengzhou University, | Zhengzhou,China | principal investigator | Steering committee |
| Yujie | Zhao | | Doctor | Zhengzhou Seventh People's Hospital, | Zhengzhou,China | principal investigator | Steering committee |
| Quanchun | Wang | | Doctor | Shenyang Fifth People's Hospital, | Shenyang,China | principal investigator | Steering committee |
| Weidong | Sun | | Doctor | Taian City Central Hospital, | Taian,China | principal investigator | Steering committee |
| Lin | Wei | | Doctor | Harbin First Hospital, | Harbin,China | principal investigator | Steering committee |
| Esther | Chan | | Doctor | The University of Hong Kong, | Hong Kong, China | principal investigator | Data Safety Monitoring Board |

Supplemental Online Content: Nonauthor Collaborators

*Indicates required information. Only first name, last name, and suffix will appear in PubMed.

| ***First Name and Middle Initial(s)** | ***Last Name** | ***Suffix (eg, Jr, III)** | Academic Degrees | Institution | Location (city, state/province, country) | Role or Contribution, eg, chair, principal investigator | Group (if more than 1 Group listed in the byline) and/or Subgroup (eg, Steering Committee) |
|---|---|---|---|---|---|---|---|
| Guangliang | Shan | | Doctor | Department of Epidemiology and Statistics, Institute of Basic Medical Sciences, Peking Union Medical College, | Beijing, China | principal investigator | Data Safety Monitoring Board |
| Chen | Yao | | Doctor | Peking University Clinical Research Institute, | Beijing, China | principal investigator | Data Safety Monitoring Board |
| Wei | Zong | | Doctor | China Foreign Affairs University, | Beijing, China | principal investigator | Data Safety Monitoring Board |
| Dandi | Chen | | Doctor | West China School of Public Health, | Chengdu, China | principal investigator | Data Safety Monitoring Board |
| Xiang | Han | | Doctor | Department of Neurology, Huashan Hospital of Fudan University, | Shanghai, China | principal investigator | Clinical events committee |
| Anding | Xu | | Doctor | Department of Neurology, the First Affiliated Hospital of Jinan University, | Guang Zhou, China | principal investigator | Clinical events committee |
| Xiaohan | Fan | | Doctor | Fuwai Hospital, Chinese Academey of Medical Sciences, | Beijing, China | principal investigator | Clinical events committee |
| Ziqiang | Yu | | Doctor | Institute of Blood Research of Jiangsu Province, | Jiangsu, China | principal investigator | Clinical events committee |
| Xiang | Gu | | Doctor | Department of Cardiology, People’s Hospital of Subei, Affiliated Hospital of YangZhou University, Jiangsu Province, | Jiangsu, China | principal investigator | Clinical events committee |
| Fulin | Ge | | Doctor | Department of Gastroenterology, Chine PLA Genral Hospital, | Beijing, China | principal investigator | Clinical events committee |
