# Supplementary material for: The Effects of Implementing a Mobile Health–Technology Supported Pathway on Atrial Fibrillation–Related Adverse Events Among Patients With Multimorbidity: The mAFA-II Randomized Clinical Trial
Source: JAMA Netw Open. 2021 Dec 21;4(12):e2140071. doi: 10.1001/jamanetworkopen.2021.40071 (PMC8693229; doi:10.1001/jamanetworkopen.2021.40071)
Supplement: Supplement 4. — Data Sharing Statement [file jamanetwopen-e2140071-s004.pdf]

Data Sharing Statement

Yao. The Effects of Implementing a Mobile Health-Technology Supported Pathway on Atrial Fibrillation-Related Adverse Events Among Patients With Multimorbidity. *JAMA Netw Open*. Published December 21, 2021. doi:10.1001/jamanetworkopen.2021.40071

Data
Data available: No
